# Quantitative Percussion Diagnostics (QPD): Seeing What the Eye Can’t With Objective Structural Data

**DOI:** 10.7759/cureus.104252

**Published:** 2026-02-25

**Authors:** Cherilyn G Sheets, James C Earthman, Gregori M Kurtzman

**Affiliations:** 1 Dentistry, Private Practice, Newport Beach, USA; 2 Bioengineering, University of California, Irvine, USA; 3 General Dentistry, Private Practice, Silver Spring, USA

**Keywords:** failing restorations, implant stability assessment, normal fit error (nfe), objective dental diagnostics, quantitative percussion diagnostics (qpd™), tooth and implant micro-mobility

## Abstract

Background

Clinical assessment of the structural integrity of teeth, restorations, and dental implants remains largely subjective, relying on radiographs, tactile evaluation, and unmeasured percussion. These methods often fail to identify early biomechanical or adhesive failure, allowing deterioration to progress asymptomatically until fracture, restoration failure, or implant complications occur. An objective, radiation-free diagnostic method capable of identifying early micromobility may improve preventive and restorative outcomes. This article is a narrative review of Quantitative Percussion Diagnostics (QPD™) and its applications in clinical practice in improving diagnostics that are not evident radiographically or with standard examination methods.

Methods

This review describes the clinical principles and applications of an FDA-cleared diagnostic system utilizing QPD™ to measure overall and internal mobility of teeth and implants. The system applies a brief, controlled mechanical impulse and analyzes the resulting response using two quantitative metrics: a mobility score reflecting overall micromobility of the site, and the normal fit error (NFE) reflecting localized internal micromobility of the site. Representative clinical cases demonstrate applications in crack detection, evaluation of failing restorations, implant stability assessment, monitoring of osseointegration, periodontal screening, occlusal overload assessment, and post-endodontic evaluation.

Results

As reported in over 30 peer-reviewed multidisciplinary studies in dental, engineering, and computer science journals, QPD™ identifies early structural compromise, including cracks, adhesive failure, internal restoration breakdown, implant component instability, and early biomechanical overload, that was not evident on routine radiographic or clinical examination. Quantitative measurements enabled baseline establishment, longitudinal monitoring, and objective confirmation of treatment outcomes following restorative, occlusal, periodontal, and implant interventions. Incorporation into routine examinations and hygiene visits was feasible without increasing chairside time.

Conclusions

QPD™ provides an objective, reproducible, and radiation-free approach for evaluating the structural integrity of teeth, restorations, and implants. This is based on decades of research from broad interdisciplinary teams of dentists, engineers, data scientists, and statisticians. By detecting early micromobility before the onset of clinical symptoms, this technology supports a preventive diagnostic paradigm, facilitates minimally invasive intervention, enhances patient communication, and contributes to improved long-term restorative and implant success.

## Introduction

Dental diagnostic methods for measuring the structural integrity of teeth and implants have remained largely subjective despite advances in restorative materials and imaging. Often, the only means of assessing a tooth or implant’s structural health is dictated by vision-generated data, such as in radiographs, transillumination, or the Miller Scale of tooth mobility. For implants, it is common to listen to the auditory sound made by tapping on the top of an implant. Data gathered from qualitative methods are often variable from clinician to clinician or even with the same clinician. By the time the damage is visible, it is usually at the end of the cycle of deterioration from fatigue failure. Examples of structural failure are fractured teeth, loose crowns, and damaged restorations. However, accurate assessment of tooth and implant integrity remains central to long-term clinical success.

The InnerView® system (Perimetrics, Redmond, WA) is based upon years of research and development, including numerous multidisciplinary studies, conducted under IRB blinded protocols with rigorous statistical analysis. Over 30 peer-reviewed articles have been published in internationally recognized journals. The scope of research has covered in vitro [1,2], in vivo [3], and finite element analysis [4]. The studies have been at independent sites across the United States and have been evaluated by the FDA for the accuracy of the results.

The FDA cleared the research version of this technology, the Periometer, in 2008. The clinical version, InnerView, was cleared in 2023 for the ability to measure the overall mobility in teeth and implants and again in 2025 for the ability to measure the internal mobility in teeth and implants caused by microgap defects. Microgap defects are caused by cracks in teeth, loose restorations, and deteriorating restorations. The patented technology called Quantitative Percussion Diagnostics (QPD™) has over 78 global patents [1,2]. The Perimetrics Database of damaged teeth includes over 1.6 million energy return graphs (ERGs), 100s of verified finite element models, 100s of replica cracked tooth studies, 100s of 3D-printed teeth to simulate other defects, and over 2,000 in vivo clinical disassemblies conducted under the clinical microscope with video and written documentation. It is the largest database of damaged teeth in the world.

QPD™ provides a radiation-free, fast, objective method of measuring the structural integrity of restorations, teeth, or implants. The technology provides a cost-effective method for diagnostics when a cone beam computed tomography (CBCT) is not available in the practice, as an adjunct to a CBCT scan, and as an easier method allowing the patient to remain in the operatory chair, eliminating the treatment disruption required to move the patient to another room to capture a scan. In less than two minutes for a full-mouth test, the clinician can see quantitative results relating to the micromobility present in the patient’s mouth. The system collects this data by means of an ERG® that then gets analyzed by two different algorithms. The first algorithm is the loss coefficient, a standard engineering algorithm, used to create the mobility scale that quantifies the overall mobility of the site. Overall mobility relates to bone quality, bone quantity, and, for implants, the osseointegration quality. The second algorithm is the normal fit error (NFE) [4]. NFE relates to the internal mobility of the site caused by the oscillations of microgap defects in the structure, such as cracks and deteriorating restorations. It is possible to identify restorative adhesive failure, crack formation, and other symptoms of structural failure long before clinical symptoms appear. This article reviews InnerView’s® operating principles, clinical applications, and advantages compared to conventional diagnostic technologies. The tools used in the case examples were purchased by those practitioners and were utilized in their clinics, similar to other dental instruments used for various dental treatments. Information (i.e., NFE and mobility) values and the resulting scales are part of the InnerView® system. This article is a narrative review of QPD and its applications in clinical practice in improving diagnostics that are not evident radiographically or with standard examination methods.

## Materials and methods

How InnerView® and QPD™ work

**Figure 1:**
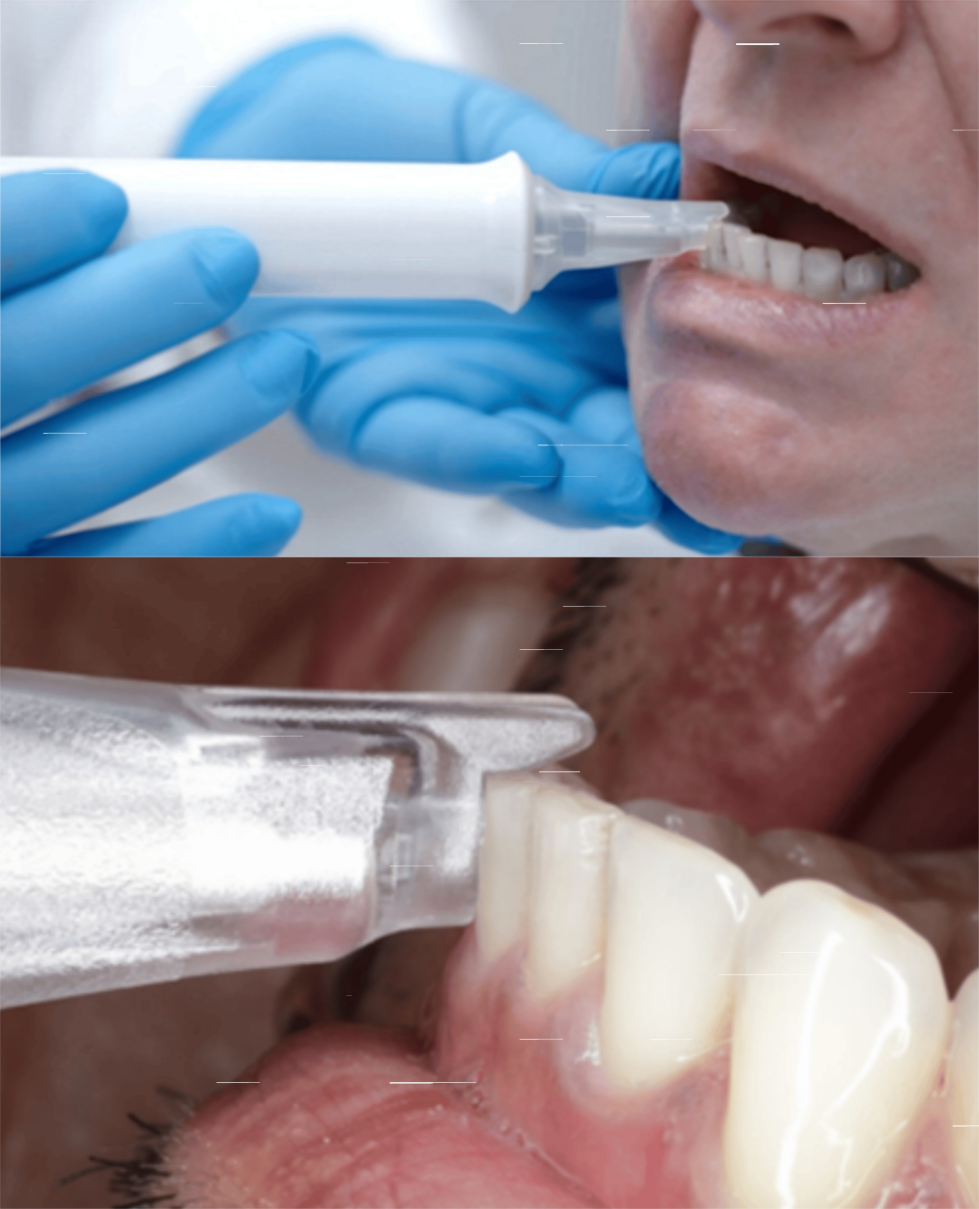
The device must be held horizontal to the floor. The handpiece tab is placed on the incisal or occlusal surface with the face of the tip flush against the buccal surface of the tooth. The patient’s head is adjusted to maintain the horizontal position of the handpiece

InnerView® and the QPD™ system deliver a precisely controlled 2.5-millisecond mechanical impulse through a disposable smart tip placed flat on the buccal surface of the tooth and stabilized by a tab placed on the occlusal or incisal surface of the tooth or implant (Figure 1). The sensitive sensor at the tip of the rod not only provides a mild percussive tap but also captures the returning energy waveform, which is then analyzed in the software by the proprietary algorithms. A complete scan of the dentition takes less than two minutes, allowing a quick assessment of the entire mouth without lengthening chairside time. In the case of restored implants or teeth, the test can be performed without prosthesis removal, so the process is simple since there is no need to disassemble. The tools used in the case examples were purchased by those practitioners and were utilized in their clinics, similar to other dental instruments used for various dental treatments. Information (i.e., NFE and mobility) values and the resulting scales are part of the InnerView® system. Because CBCT-based detection of dehiscence and fine defects has known limitations, a formal validation protocol comparing QPD outputs to imaging and surgical/gold-standard findings is required [5].

The initial testing on a patient serves two purposes: it highlights areas of interest that need further evaluation, and it provides a baseline for future measurements. The system also provides a monitoring report that tracks the mobility and NFE scores over time. This creates a structural health history for every tooth and implant in a patient’s mouth. As patients are seen in the hygiene room on recall, this short two-minute test adds data to the monitoring of the patient’s structural health and frequently locates areas of breakdown that are asymptomatic. Finding these asymptomatic beginning pathologies saves patients pain, money, and tooth or bone structure by allowing early preventive or proactive treatment. 

**Figure 2:**
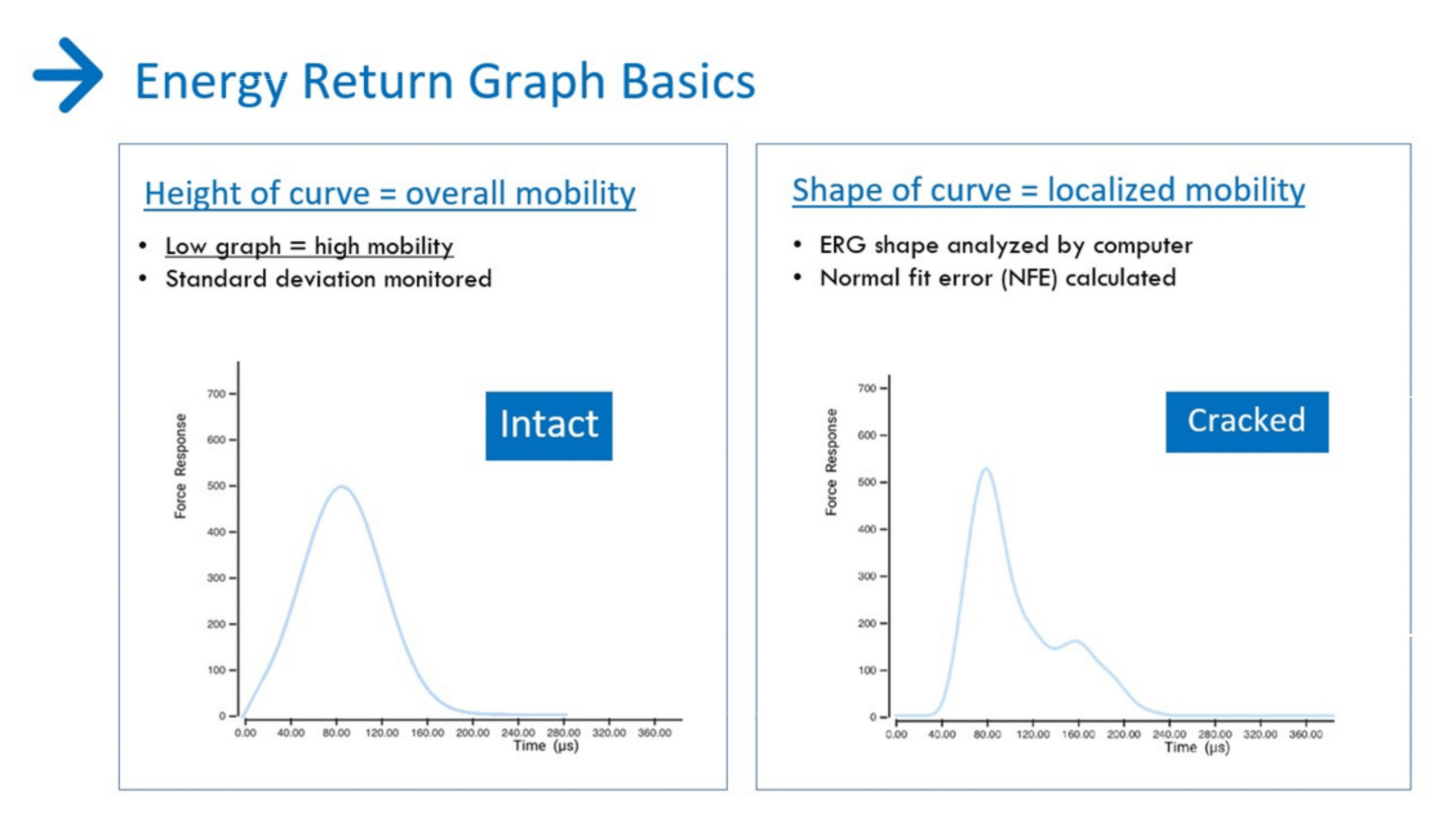
The height of the ERG relates to how much overall micromovement is in the site. The shape of the ERG relates to localized movement in the site caused by cracks, defective restorations, and other microgap defects that oscillate ERG: energy return graph

Mobility testing is on a scale of 0 to 100. The average values for teeth are usually found in the 58-79 range. The higher the reading, the more micromobility. The lower the reading, the denser the bone. Teeth with very low mobility scores could have ankylosis. The peak height on the ERG determines the mobility score. The higher the peak, the lower the micromobility. A perfectly shaped bell curve is the structural fingerprint of a structurally healthy site (Figure 2, left).

**Figure 3:**
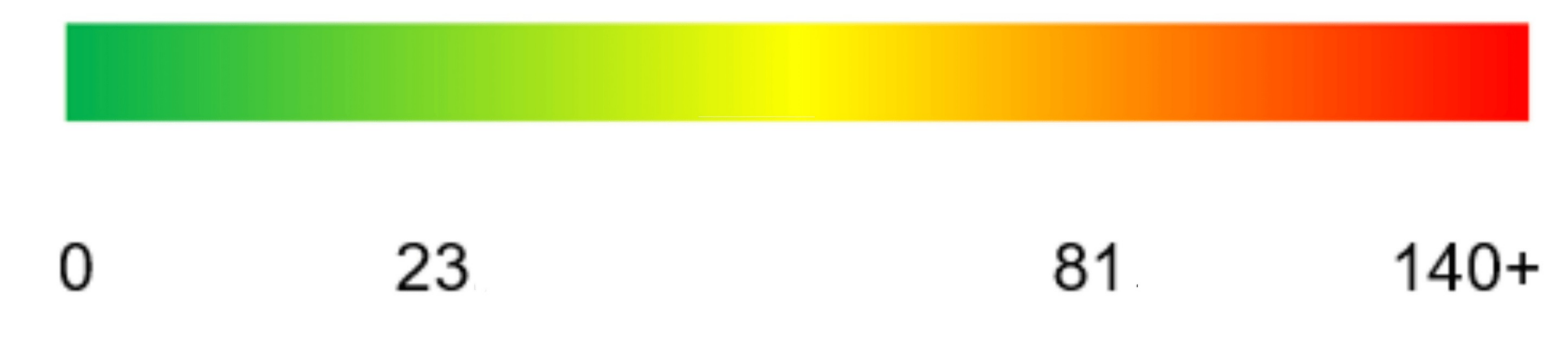
Color scale showing the increasing severity as the NFE score increases (InnerView software) NFE: normal fit error

The NFE is determined by the shape of the ERG. NFE becomes greater as the shape of the ERG deviates more and more from this ideal shape as a result of increased damage at the site (Figure 2, right). The NFE scale is from 0 (associated with an intact structure with no structural abnormality) to 140+ (indicating severe localized micromovement) (Figure 3). Specific ranges for each tooth geometry are provided to the clinician. The basics are that the ideal shape of an ERG is the bell-shaped curve. The more peaks and valleys, and the further out in time it takes for the oscillations to stop, the more damaged the site. 

## Results

Clinical applications

Crack Detection in Teeth

Cracks within the enamel or dentin often develop and progress silently and, therefore, are very difficult to diagnose until pain or a visible fracture occurs. Standard methods, such as transillumination, dyes, or radiographs, detect only surface or gross defects. Percussion tests without measurement are purely subjective and are not clinically helpful in identifying early asymptomatic cracks. Radiographically, the crack needs to be oriented perpendicular to the radiograph sensor and deep enough in the tooth structure to be identifiable radiographically. CBCT imaging has been utilized to identify cracks, but lacks the resolution to visualize fine internal fractures, and is only helpful when the crack has become severe or catastrophic. QPD™ detects early structural changes by measuring alterations in oscillation response long before cracks are visible [6]. By identifying compromised responses early, clinicians can intervene with conservative restorations or cuspal coverage before catastrophic tooth failure occurs.

Case Example: Large Existing Amalgam Restoration

**Figure 4:**
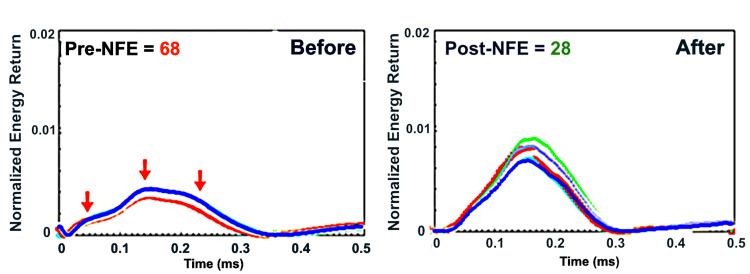
Pretreatment testing of a tooth with a large amalgam and posttreatment testing demonstrating marked improvement in NFE NFE: normal fit error

An old amalgam filling with minor marginal stains presented on tooth #29 (mandibular left 2nd premolar). The tooth was asymptomatic with outward signs of potential microleakage (Figure 4).

QPD™ testing identified a pre-NFE reading of 68. Following the removal of the restoration, problems became visible, confirming microleakage under the restoration, which allowed undetected decay to advance closer to the pulp. The unsupported tooth structure had developed enamel cracks. The tooth was restored with a bonded core buildup and crown. QPD™ testing occurred following restoration to establish a new baseline measurement for the tooth. The posttreatment NFE reading of 28 confirmed that there was improved stability of the tooth.

Detection of Failing Restorations

Adhesive breakdown, marginal leakage, or internal voids can develop beneath apparently intact restorations. Explorers and radiographs are limited in detecting subsurface issues and usually reveal deterioration only after recurrent decay or marginal breakdown appears. The InnerView® system objectively identifies subtle losses in stiffness or changes in energy dissipation associated with early debonding or microleakage [3,7]. Detecting failure before clinical symptoms arise allows targeted conservative treatment rather than extensive restorative procedures, preserving sound tooth structure and reducing unnecessary intervention.

Case Example: Large Amalgam Restoration With Beginning Cold Sensitivity

**Figure 5:**
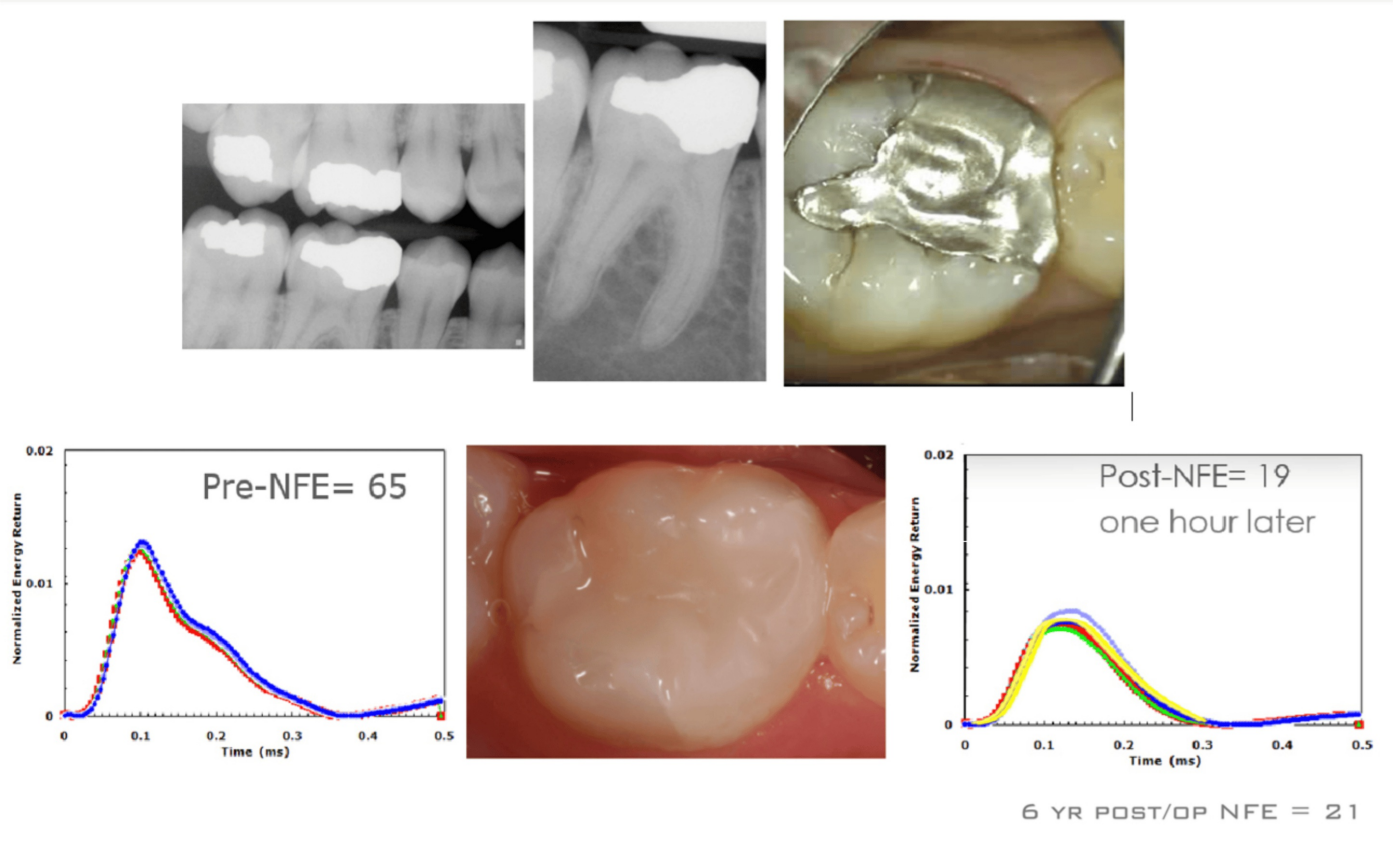
Large molar amalgam restoration with cold sensitivity and no radiographic issues. The pre-NFE of 65 indicated problems. The immediate composite restoration measured a post-NFE reading of 19, and symptoms were eliminated NFE: normal fit error

The patient had recently begun noticing light cold sensitivity on her right mandibular first molar. Radiographic examination noted no visible pathology (Figure 5, top). QPD testing noted an elevated NFE of 65. It was decided with the patient to remove the restoration even though there was no visible problem. During restoration removal, a significant crack appeared in the restoration, which then easily fractured away, exposing significant moisture and decay due to microleakage. Upon removal of the amalgam, the decay covered the entire pulpal floor, extending deep into the body of the tooth. Upon removal of all the decay, a crack in the pulpal floor was visible that extended halfway from the distal to the mesial. The tooth was restored with a bonded composite as an interim restoration. Immediately after the restoration was completed, the tooth was tested again, and the posttreatment NFE was lowered from 65 to 19 (Figure 5, bottom). The patient moved out of the country and lost contact with the practice for six years. When she returned, the tooth was tested, and surprisingly, the NFE was still low at 21. The patient reported that the tooth had remained asymptomatic. 

Implant Stability and Early Loading Decisions

Accurate assessment of implant stability is critical for determining readiness for provisionalization or definitive loading. Clinicians have traditionally relied on insertion torque, reverse torque, tactile evaluation, or resonance frequency analysis (RFA) (implant stability quotient (ISQ)). Only ISQ provides a measured response, and then with only one metric. While useful, these methods cannot detect micromobility at the bone-implant interface of both overall and internal mobility measurements. QPD™ measures structural response directly, providing quantifiable data on implant rigidity and early osseointegration [8]. Stable readings support immediate or early loading, whereas increased mobility values prompt the clinician to delay restoration or adjust occlusal loading to prevent overloading and potential failure.

Case Example: Implant Stability Before Final Restoration

**Figure 6:**
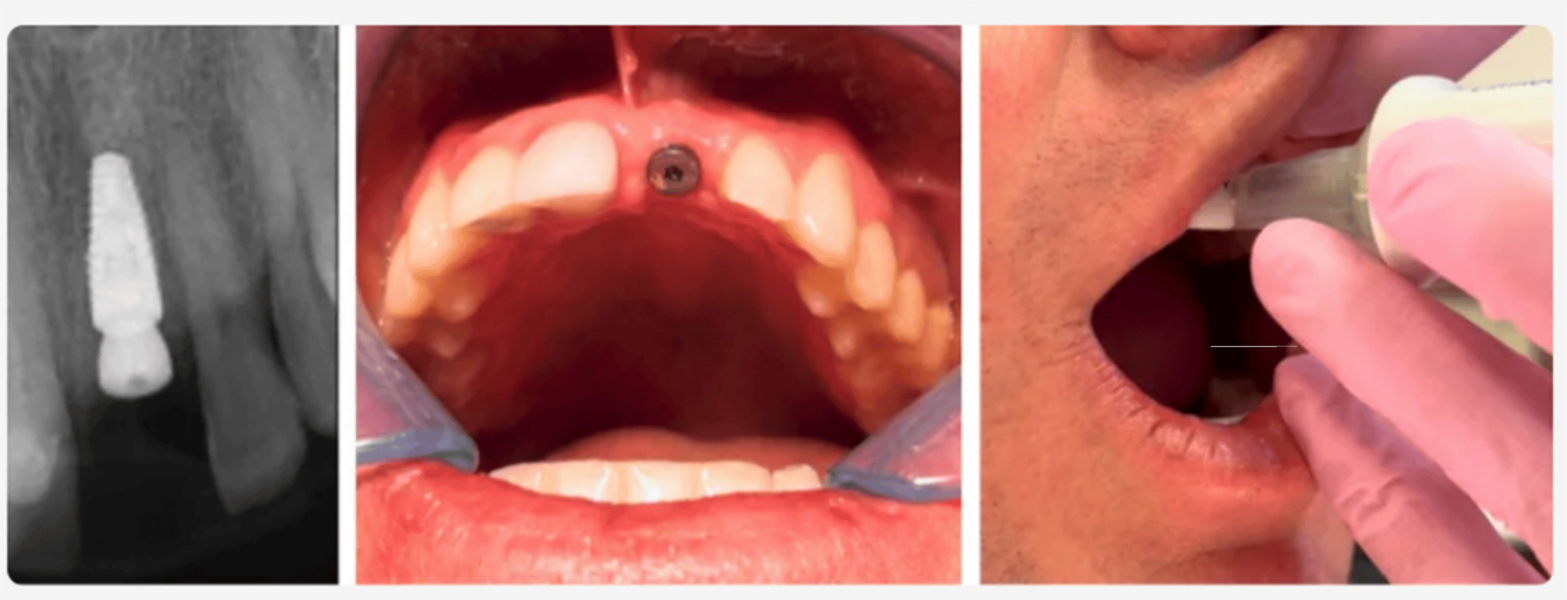
Five-month evaluation postimplant placement demonstrating radiographic bone contact along the entire implant length, healthy gingival tissue, and a stable QDP mobility score taken on the healing abutment torqued to 15 newtons QDP: Quantitative Percussion Diagnostics

A 35-year-old patient with a history of trauma and endodontic treatment to teeth #7-9 presented with a chief complaint of a loose front tooth (#9). The tooth on examination was terminal. Replacement with an immediate implant was recommended and accepted by the patient. The tooth was extracted, an implant was placed, but due to a very low insertion torque, delayed loading was indicated. A healing abutment was placed, and an Essix provisional was delivered. At five months postimplant placement, a radiograph confirmed that there was contact of bone along the entire length of the implant. Clinically, the gingival tissue was noninflamed and healthy (Figure 6). An InnerView mobility assessment at five months postsurgery confirmed the healing process was complete and osseointegration had occurred.

**Figure 7:**
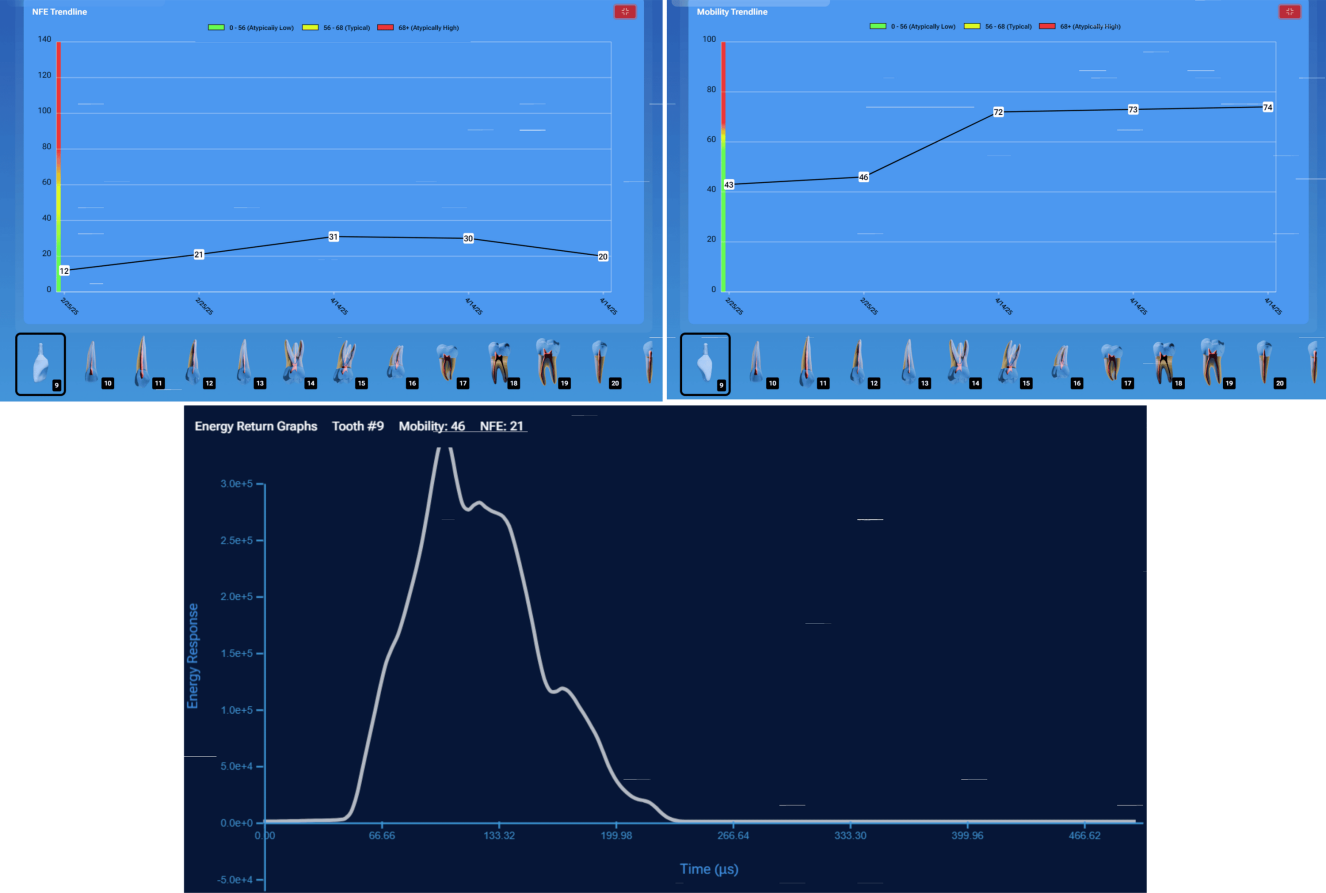
InnerView stable mobility and NFE scores (top) and a Gaussian-shaped ERG (bottom) was observed with minor shape irregularities indicating very minor localized micromovement NFE: normal fit error; ERG: energy return graph

The QPD mobility score was 46, indicating the implant had integrated well with the surrounding bone. A Gaussian-shaped curve was observed, with slight undulations suggesting minor residual instability, but not enough to warrant further delays, prompting cautious progression to temporization with ongoing monitoring (Figure 7).

Monitoring Osseointegration and Peri-implant Health

Radiographs and periodontal probing remain the standard for monitoring implants. Both are reactive indicators, revealing bone loss only after significant hard tissue resorption. Yet, subtle mechanical changes may occur long before radiographic signs appear clinically. By tracking implant mobility trends over time, InnerView® enables early identification of biomechanical stress or incipient bone loss, allowing early intervention. Deviations in the numerical trendline signal the need for periodontal maintenance or occlusal adjustment before peri-implantitis can develop [9,10]. This proactively transforms peri-implant monitoring from a reactive to a preventive process.

Case Example: Monitoring Implant Health Over Time

**Figure 8:**
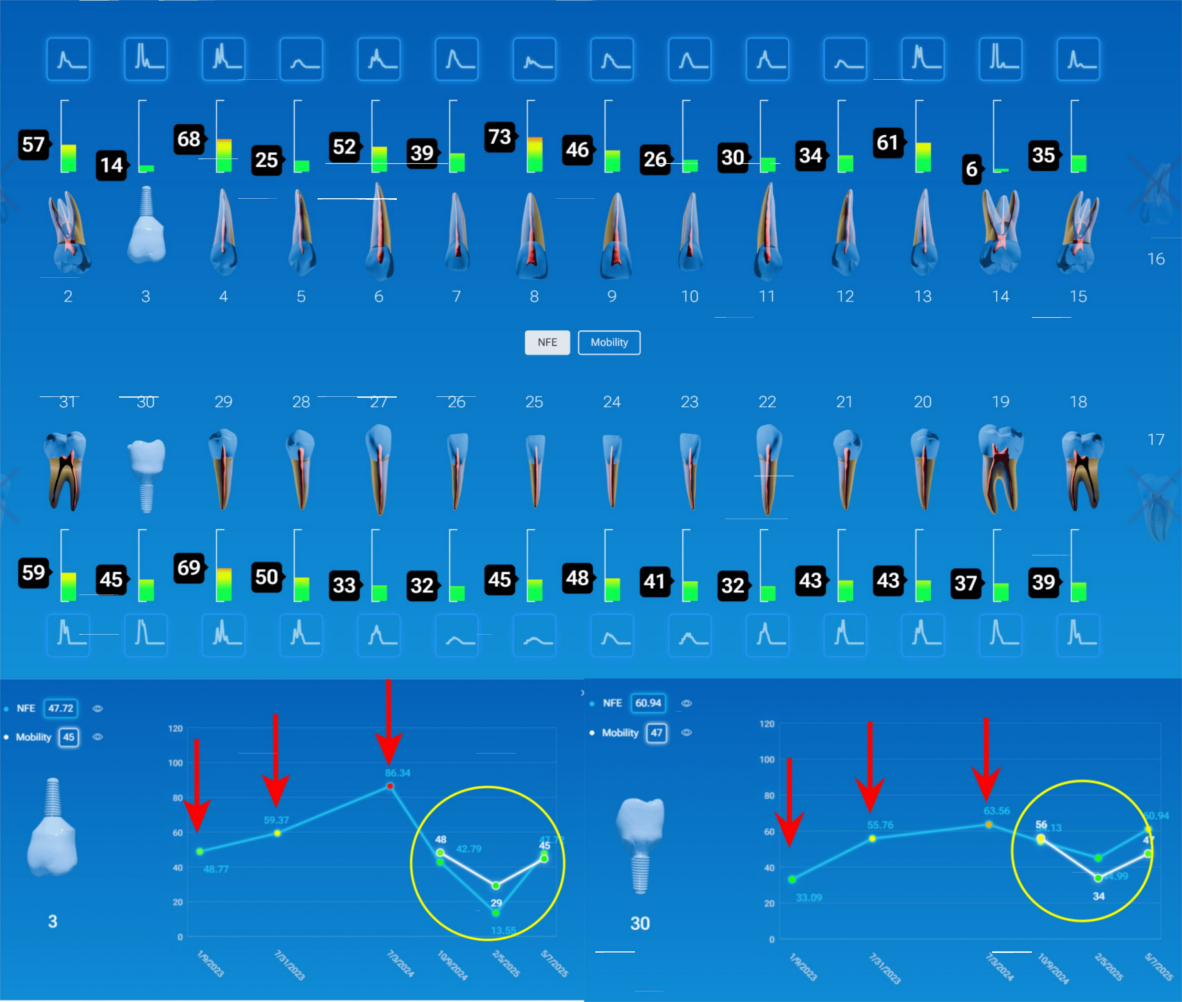
NFE values demonstrated elevating readings on implants at #3 and 30 and decreases following utilization of a custom nightguard at subsequent recall appointments NFE: normal fit error

A patient was monitored over time on her restored implants at the right maxillary (#3) and mandibular (#30) 1st molars and natural dentition. She remained symptom-free, and the implants appeared stable both clinically and radiographically. However, during hygiene monitoring testing at prophy appointments, InnerView had revealed elevated NFE scores (Figure 8).

Implant #3 demonstrated an NFE score that had gradually increased over multiple visits, signaling a potential issue. Implant #30 displayed a similar pattern, suggesting the need for intervention before structural damage occurred due to overloading the implants. Based on the data, occlusal protection using a custom nightguard to reduce excessive force on the implants was recommended and provided. Continued trendline monitoring was done to confirm the stability of the intervention. Following occlusal protection intervention, NFE scores significantly decreased, confirming improved implant stability, validating the effectiveness of occlusal protection in maintaining implant health.

Implant Structural Issues

InnerView aids in identifying implant screw loosening, abutment component fracture, or fracture of the implant itself by monitoring micromobility, as seen in the NFE score. When not caught early, screw loosening may lead to a fracture of the screw or damage to the threaded channel at the internal of the implant fixture. Fracture of the implant itself also may occur and is typically not identified until catastrophic failure results. When caught early, salvaging the implant may be possible.

Case Example: Implant Fracture at Its Connector

**Figure 9:**
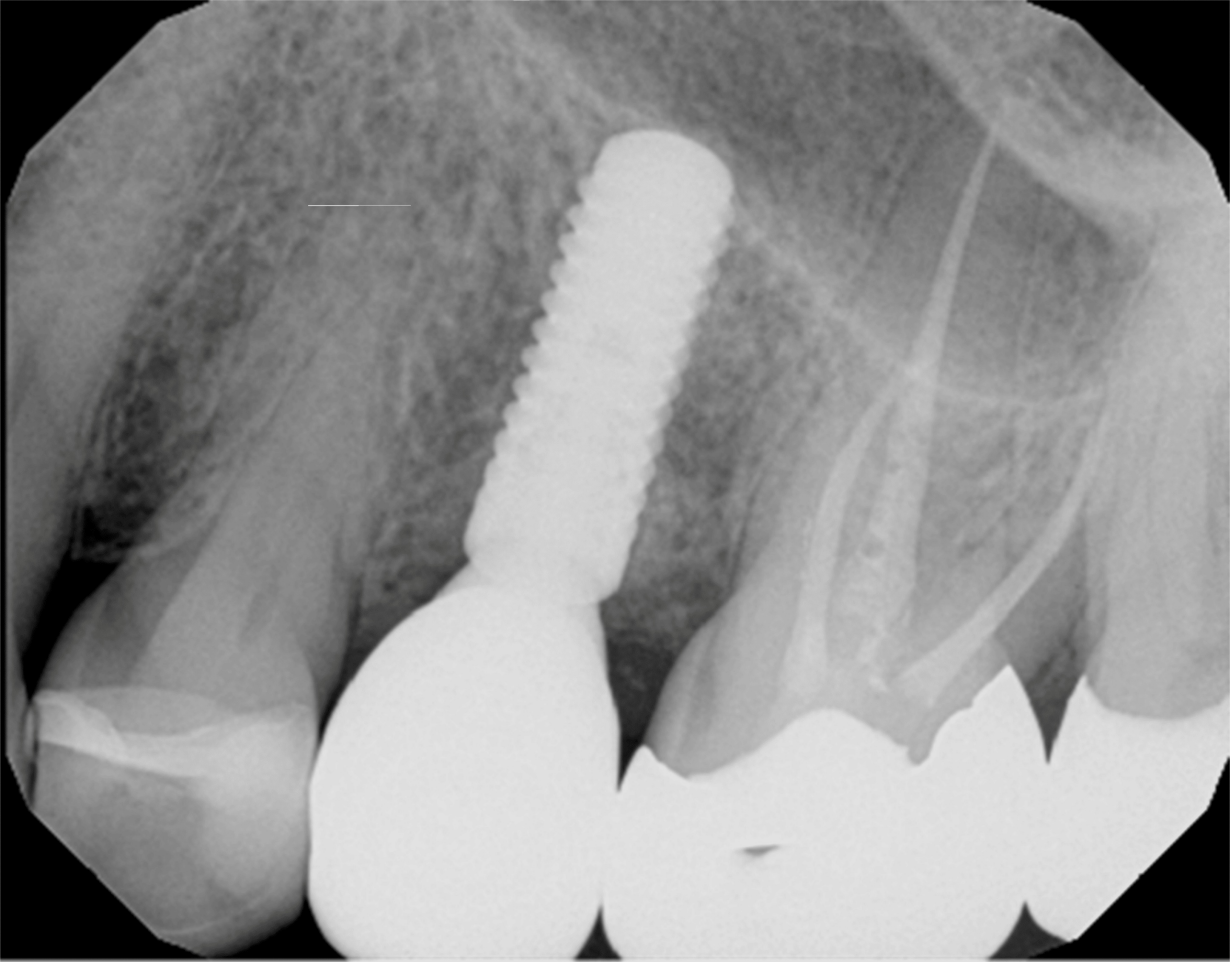
Radiograph of the implant at #13 demonstrating good bone levels and no noticeable issues

**Figure 10:**
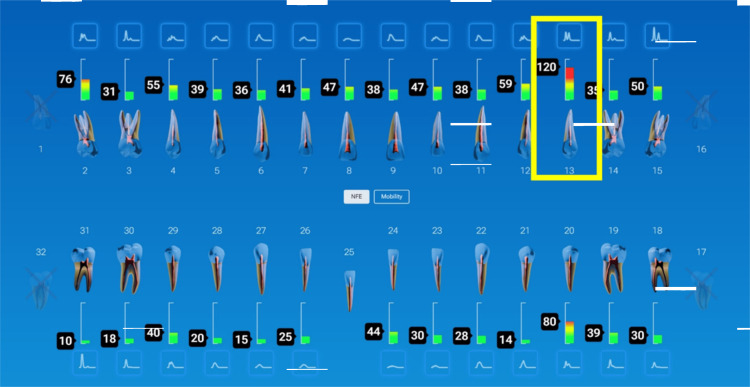
Full mouth QPD testing showed a high NFE reading of 120 on the implant at site #13 QPD: Quantitative Percussion Diagnostics; NFE: normal fit error

**Figure 11:**
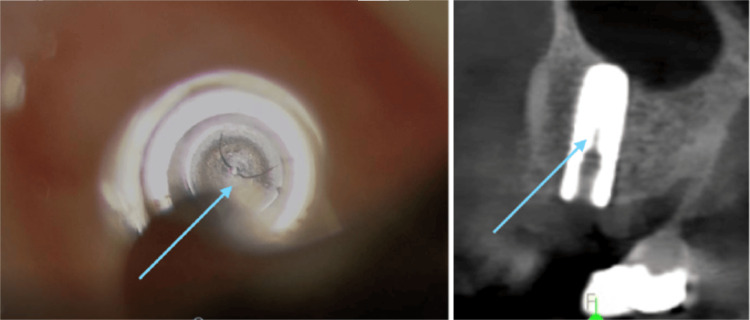
Crack noted at the base of the implants connector when examined through the clinical microscope at 16x following removal of the restoration and confirmed on the CBCT CBCT: cone beam computed tomography

A patient presented for a new patient examination with no complaints. Her one dental implant appeared very healthy radiographically and clinically (Figure 9). However, InnerView testing resulted in an NFE reading of 120. It was tested again at a follow-up appointment and was still at 119 (Figure 10). The first thought was that it must be a loose screw causing the internal mobility. But when the screw was examined, it was very tight. The screw was loosened, and the restoration was removed for examination of the implant fixture. A crack was noted at the base of the implant fixture, which may have resulted from tightening the screw beyond the manufacturer's recommended torque, placing internal pressure on the implant fixture (Figure 11, left). A CBCT was taken, confirming the extent of the fracture in the implant (Figure 11, right). The damage was internal at this point and had not extended to the implant's outer surface; there was no infection, and the implant was well-osseointegrated. Due to these factors, the clinician could replace the crown screwed to the manufacturer's recommended torque and monitor the site in the future with QPD. If this had not been discovered, it was possible that further damage to the implant’s internal connector and body could result in more advanced problems or even the loss of the implant.

Undiagnosed Structural Defects Identified With InnerView

It is challenging to identify clinical issues within existing restorations, especially with normal radiographs and no detectable marginal discrepancy. Sometimes the first sign of trouble is the failure of the restoration. But catching problems early may allow early preventive or proactive treatment rather than extensive restorations, endodontic treatment, or possibly even extraction. QPD and its associated Mobility and NFE readings aid in earlier identification of failing crowns, onlays, post/cores, or underlying core buildups before the patient notices any clinical symptoms.

Case Example: Early Detection of a Failing Crown

**Figure 12:**
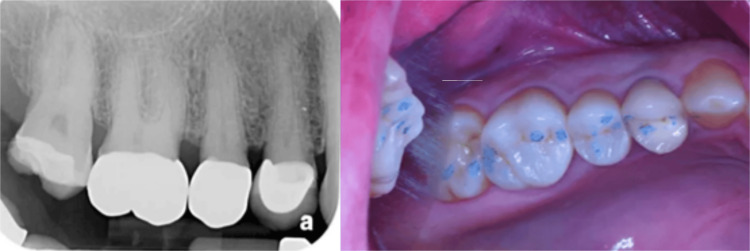
Radiograph and clinical picture of previously restored teeth in the upper right quadrant

**Figure 13:**
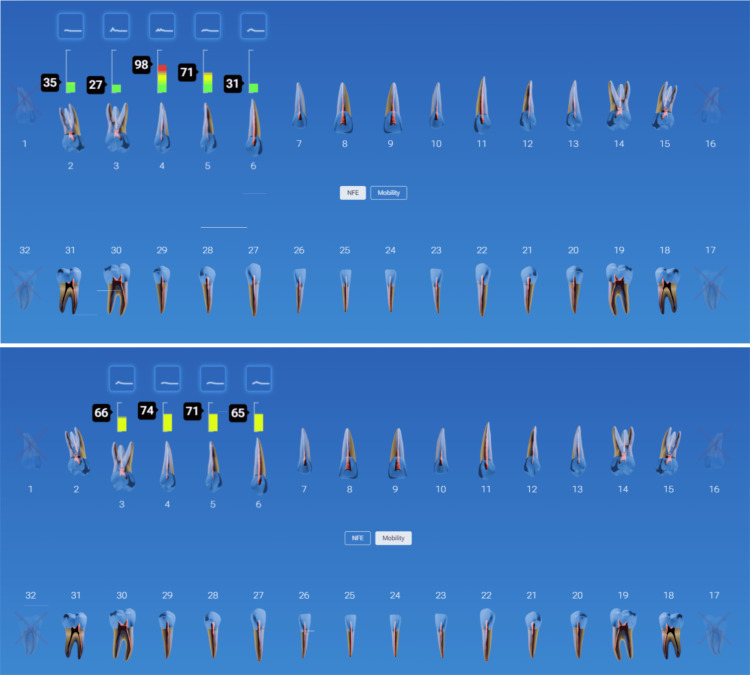
QDP testing of the upper right quadrant for NFE (left) and mobility (right) demonstrating high NFE values on teeth #4 and 5 NFE: normal fit error; QDP: Quantitative Percussion Diagnostics

**Figure 14:**
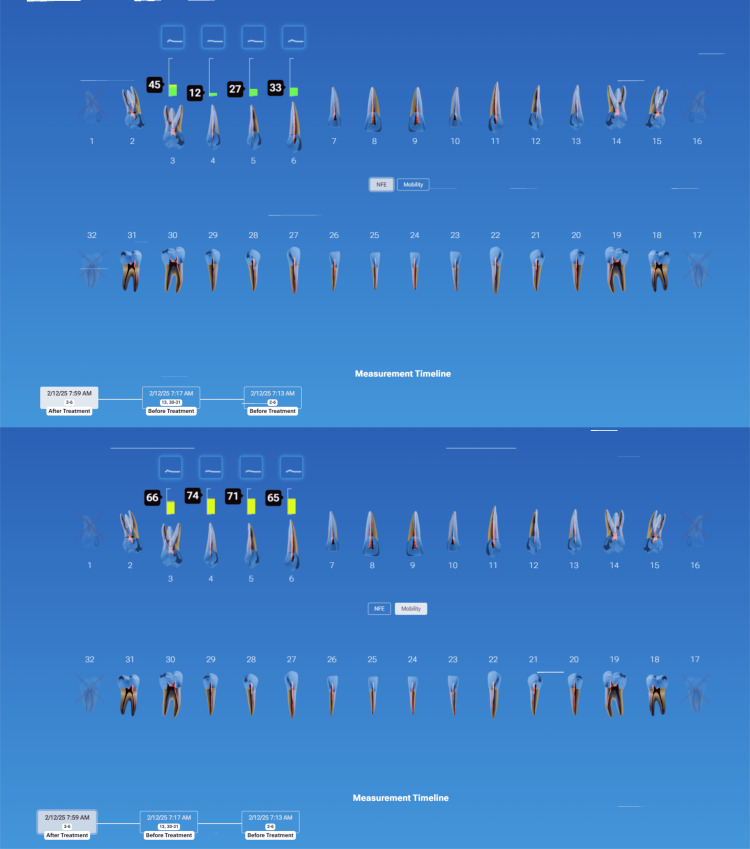
NFE readings following treatment demonstrating reduced values of teeth #4 and 5 bringing them into normal values NFE: normal fit error

During examination of a patient with previous crowns on teeth #3-5, a radiograph noted periapical pathology at the apex of tooth #2 and decay marginally on the crown at #4, with nothing noted on crown #5 (Figure 12). Traditional diagnostics developed a treatment plan to endodontically treat tooth #2, followed by a post/core and crown, and replacement of the crown on tooth #4. QDP testing was done with InnerView, and high measurement values confirmed that the planned crown replacement on #4 was necessary (Figure 13). Unexpected structural instability was detected on crown #5, despite appearing normal on X-rays and clinical exam. InnerView identified hidden defects that conventional diagnostics would have missed, prompting a more cautious and proactive treatment approach. Upon disassembly of crown 5, it was discovered that the core buildup was loose. and replacement of the core buildup and crown on #5 was indicated. Therefore, the high NFE numbers for tooth #4 confirmed a need for replacement of that failing crown, and on #5, replacement of the core buildup and replacement of the crown. Following treatment retesting with InnerView, the post-NFE #4 mobility readings significantly decreased, confirming that the new restoration had stabilized the tooth, and on #5, which had shown the hidden mobility, now demonstrated structural integrity with the new core buildup and crown replacement (Figure 14).

The Importance of Hygiene Monitoring With QPD: Identification of Periodontal Issues During Prophy Appointments

As outlined, structural integrity issues are often asymptomatic, and traditional diagnostic aids (radiographs and visual examination) do not identify these problems until late in the damage cycle. QPD testing can aid in identifying clinical issues during routine recall hygiene appointments or initial new patient appointments. Full-mouth QPD testing can be performed by the hygienist in less than two minutes, thus not lengthening the appointment time. Hygiene monitoring also helps identify asymptomatic clenchers or bruxers. InnerView is a mechanical test based on physics and math that shows what happens when your patient is eating and bruxing, and the more they have parafunctional habits, the greater the odds that there will be a structural breakdown.

Balanced occlusion is fundamental to the tooth/implant and restorative longevity, yet assessing occlusal stress objectively has long been a challenge. Conventional techniques rely on articulating paper or patient feedback, which provides qualitative but not quantitative information. QPD™ enables direct comparison of mechanical stiffness between teeth or implants, highlighting overloaded units that show elevated micromobility. Guided occlusal adjustment based on these measurements helps redistribute load, prevent crack propagation, and extend the service life of both restorations and implants.

Case Example: Incorporating QPD Testing Into a New Patient Examination

Clinicians often wonder how to incorporate QPD testing into a busy office workflow. It is recommended that a full InnerView test be taken by the staff along with the other records standard for a comprehensive examination by the doctor, i.e., radiographs, photographs, scans, etc. This next case is a good example of why it is important to have the structural data ready to be evaluated prior to even looking into the patient’s mouth. 

**Figure 15:**
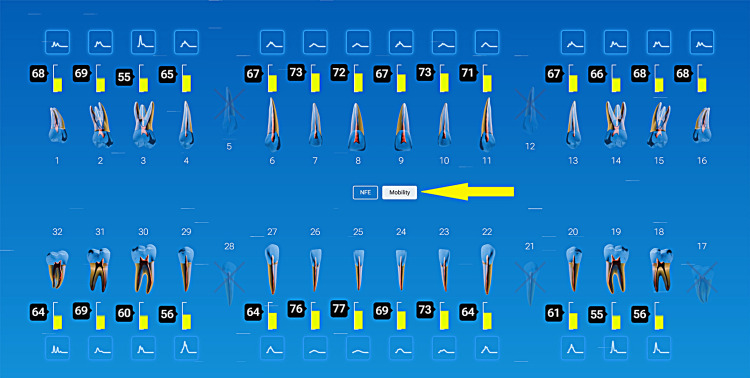
Mobility readings of the dentition

**Figure 16:**
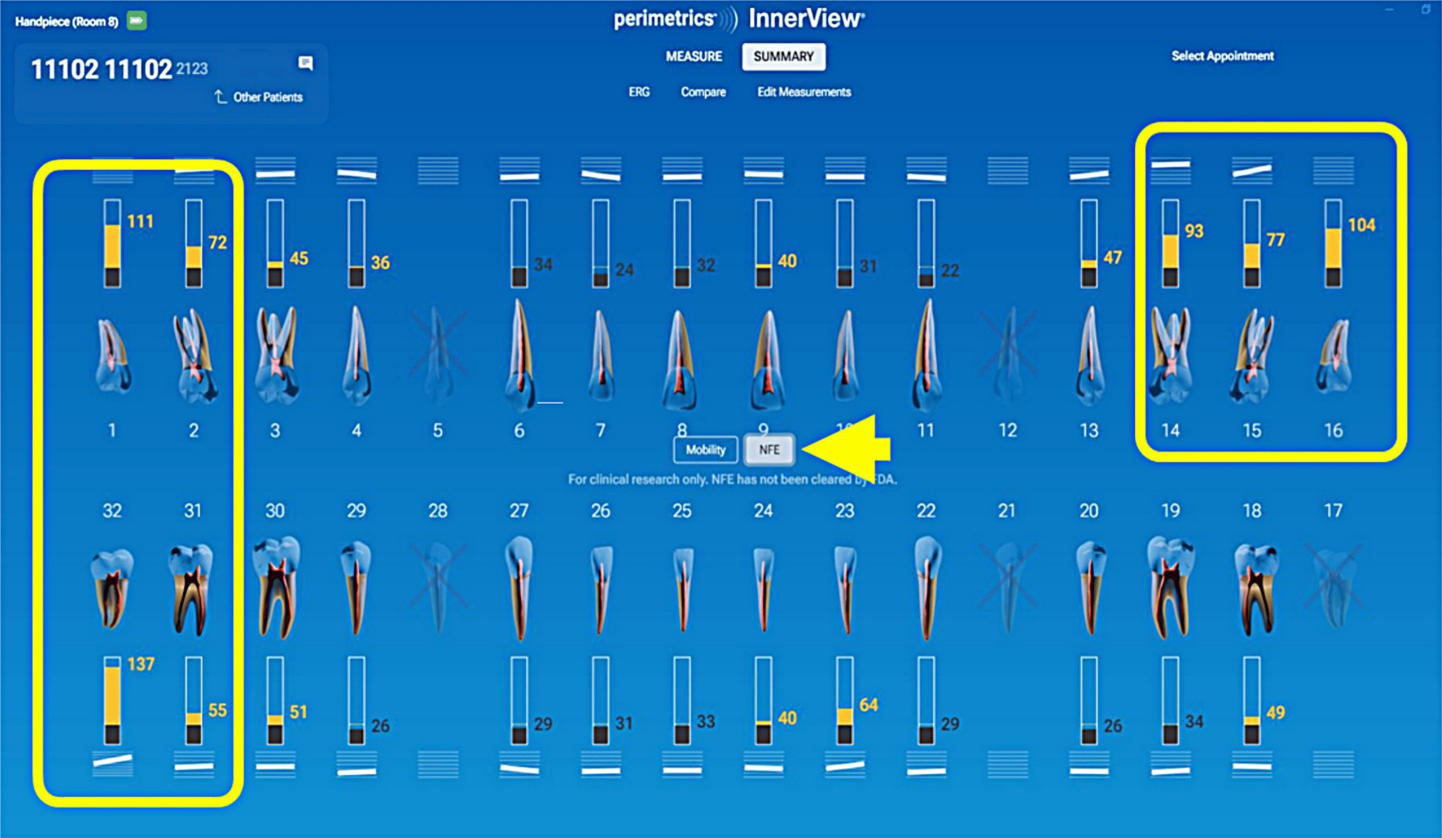
NFE readings of the dentition of a new patient highlighting teeth with high values NFE: normal fit error

A patient presented for an initial exam as a new patient. Prior to looking into the mouth, the QPD results were evaluated and shared with the patient. The mobility scores looked within the normal range (Figure 15), but the NFE scores had some very elevated readings for the maxillary left quadrant and mandibular right quadrant (Figure 16). Those sites now have the attention of the clinician and patient. Before the examination is over, you would hope to have an explanation for the high readings.

The highlights of the comprehensive examination were that tooth #16, which had a reading of 104, was extruded and in constant occlusal trauma. Tooth #14 had a small amalgam filling on the occlusal, but it also had a strong gray coloration on the buccal due to the extent of the restoration beneath the occlusal alloy. Tooth #30 had a fractured ML cusp, eliminating a piece that would have oscillated when percussed, but still had an NFE of 51, higher than a lot of his other mandibular teeth, indicating there were remaining defects. The mandibular right posterior quadrant had significant bone loss and periodontal disease with elevated NFE readings indicating reduced bone support. 

**Figure 17:**
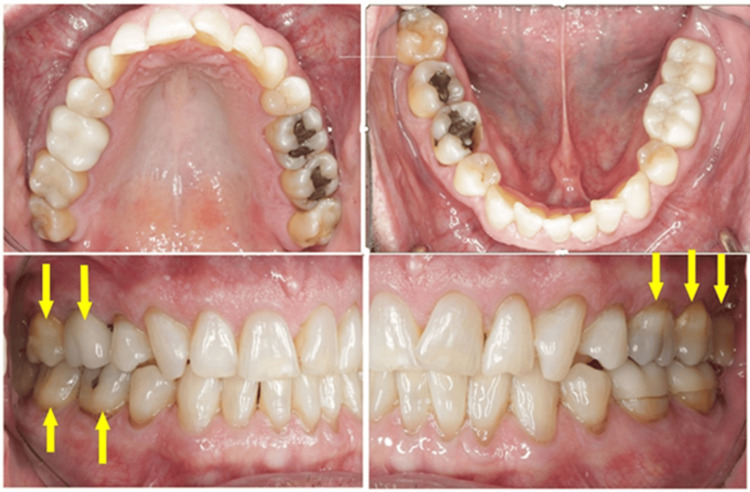
Clinical appearance of the dentition was not highly damaged. Yellow arrows indicate teeth with high NFEs. Only structural defect visible was #30 fractured ML cusp NFE: normal fit error; ML: mesiolingual

**Figure 18:**
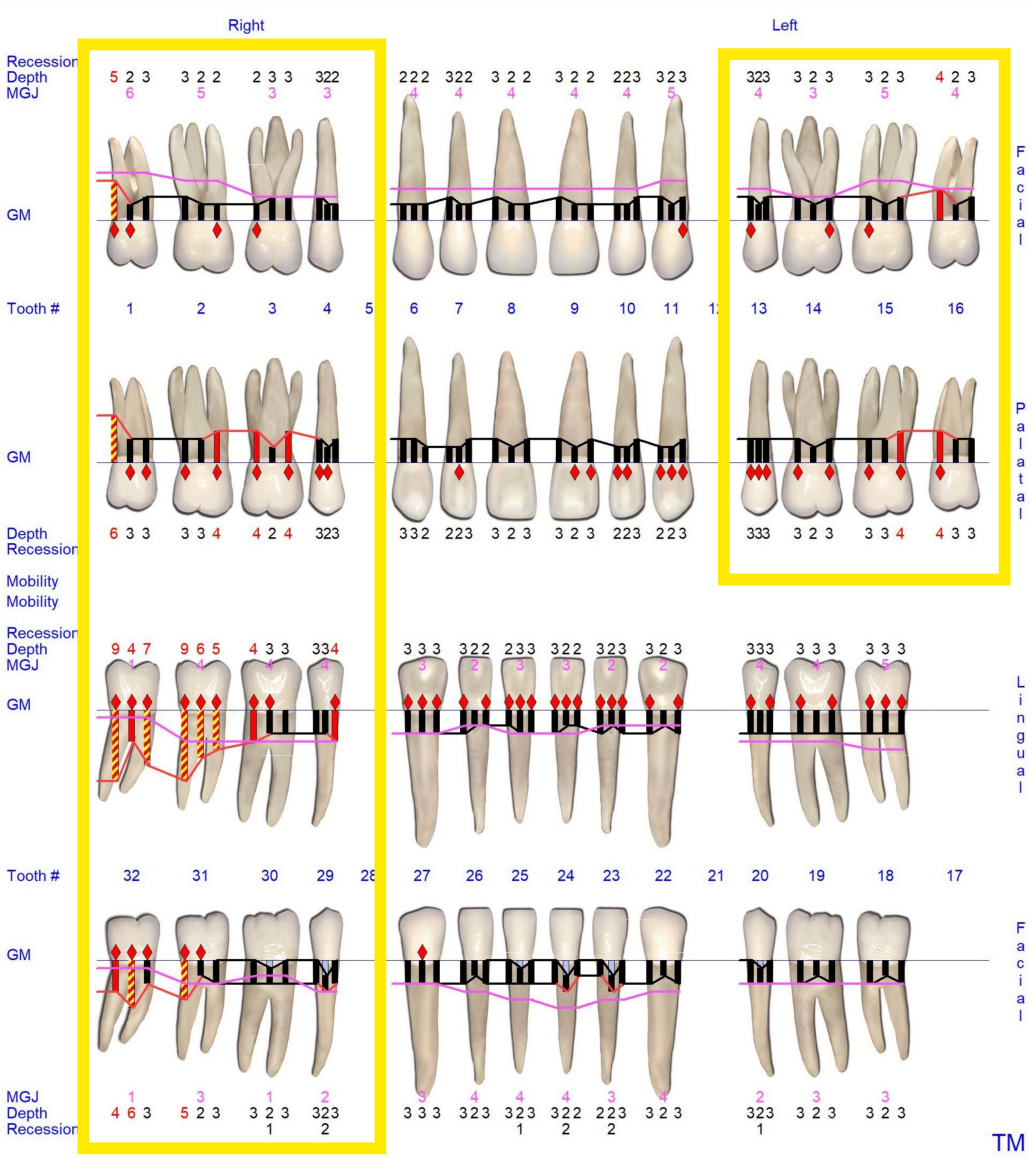
Periodontal charting of the dentition demonstrating deep probing on many of the teeth with high NFE readings NFE: normal fit error

Now armed with structural data, you can start asking more questions about habits. For instance, the patient is a clencher/bruxer, an ice chewer, and lifts heavy weights professionally. There are visible micro/macro cracks in many teeth (Figure 17). Mobility readings showed normal to high bone density, so many of his problems were internal mobility from cracks and fractures. Periodontal charting was performed (Figure 18), and bleeding with deeper probing was noted on teeth #1, 4, 31, and 32, with bone loss on teeth #30-32. There was heavy generalized subgingival calculus and open margins on fillings, and so periodontal therapy was recommended and initiated. The patient had no current TMD issues, but had numerous signs of heavy bruxism, occlusal interferences, and micro- and macrofractures of teeth, and rated his stress as high.

What do you do with this information? Do you start crowning everything? A more measured approach is to start with the periodontal disease elimination treatment and the extraction of the hyper-erupted tooth #16. Additionally, while his tissues are healing, place a provisional composite restoration to stabilize tooth #30 and make an athletic guard for when he is lifting heavy weights. Sequencing treatment this way will allow monitoring with QPD to see the results of this initial treatment and continue to educate your patient about the causes of structural breakdown. 

The patient is now starting to understand what the QPD measurements mean and even seeing areas of improvement or continuing breakdown. Once all the preventive and proactive treatments are completed, a final treatment plan can be established for phase II treatment to correct any remaining structural problems. InnerView QPD monitoring reports would continue to be generated as treatment progressed and at hygiene recall appointments to further monitor and educate the patient. 

Post-endodontic Integrity

Endodontically treated teeth restored with posts and cores often fail from post debonding, leading potentially to vertical root fracture. These conditions are rarely detected radiographically until catastrophic failure occurs. Conventional testing methods offer little diagnostic value in identifying these early changes. The InnerView® system detects internal oscillation differences that indicate subtle instability within the restored tooth. Recognizing those mechanical weaknesses early enables reinforcement or retreatment before complete failure of the restoration or root structure.

Case Example: Detecting a Loose Post/Core Under a Crown

A patient treated in the practice 10 years prior with crowns on the maxillary arch had relocated to another state. The patient had recently been seen by a periodontist who noted inflammation around tooth #7. Surgery eliminated the pocket but created an aesthetic problem.

**Figure 19:**
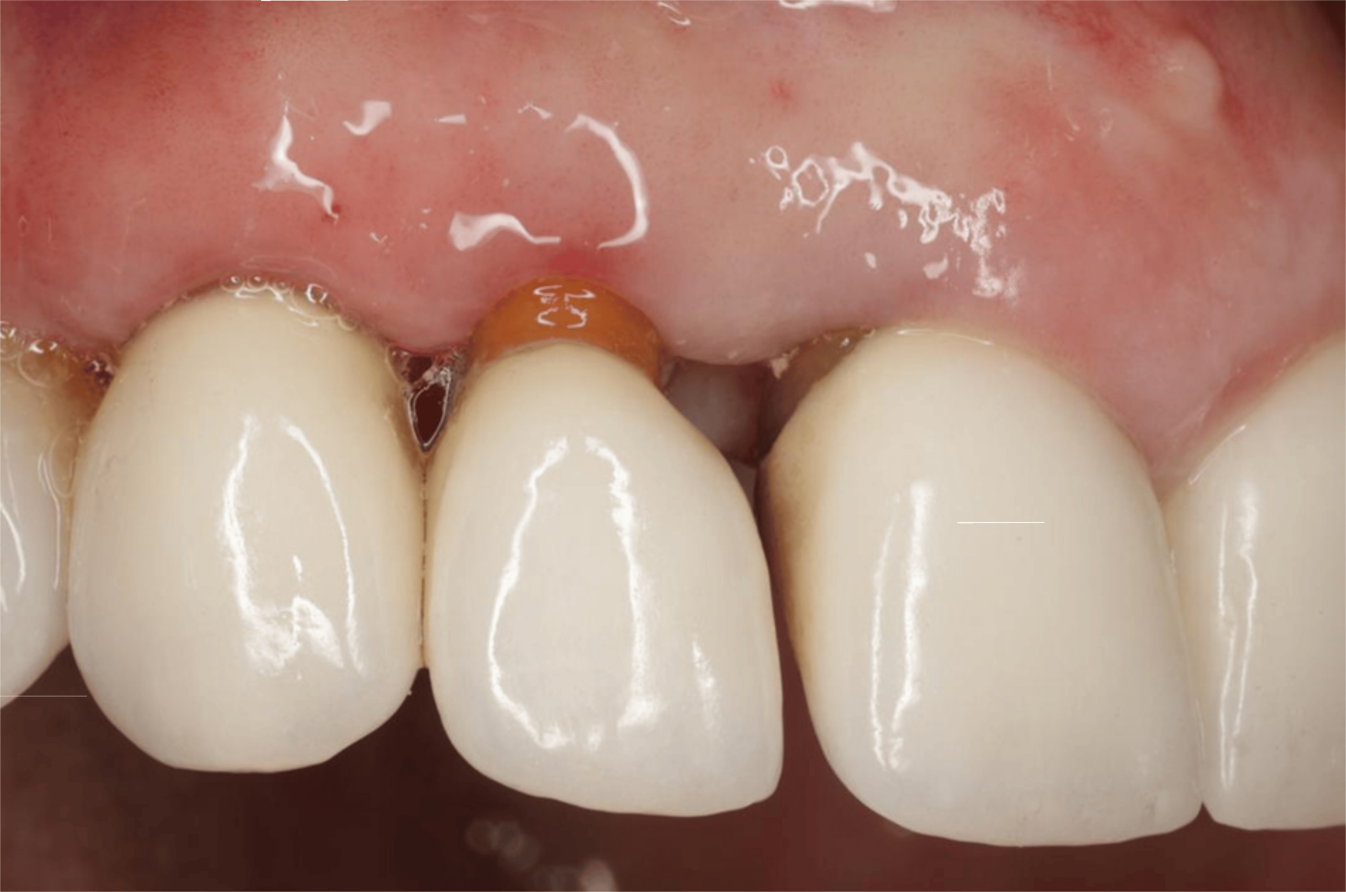
Tooth #7 following periodontal surgery and prior to removing the crown for aesthetic reasons

The patient returned to the original practice to discuss replacement of the crown on tooth #7 to correct aesthetic concerns (Figure 19). A short-term solution was suggested to place a provisional crown to close the spaces resulting from bilateral loss of the papilla until a final restoration could be performed. Radiographically, no pathology was noted.

**Figure 20:**
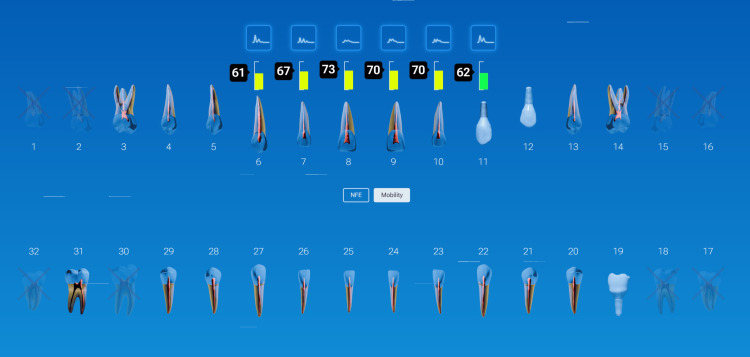
InnerView testing demonstrating normal mobility on the teeth

**Figure 21:**
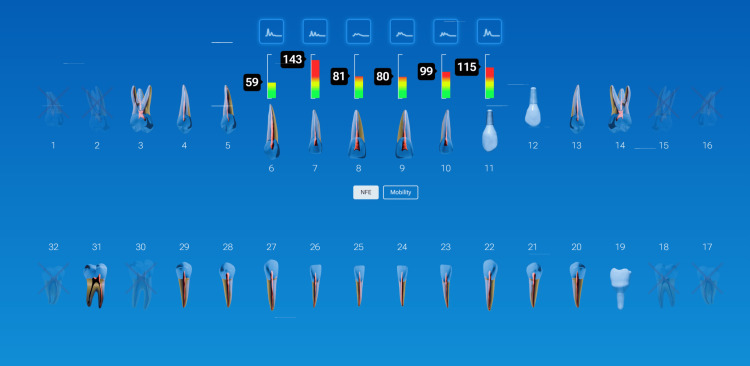
InnerView NFE test scores demonstrated a very elevated and abnormal reading on tooth #7 at 143. Other high readings were also noted NFE: normal fit error

**Figure 22:**
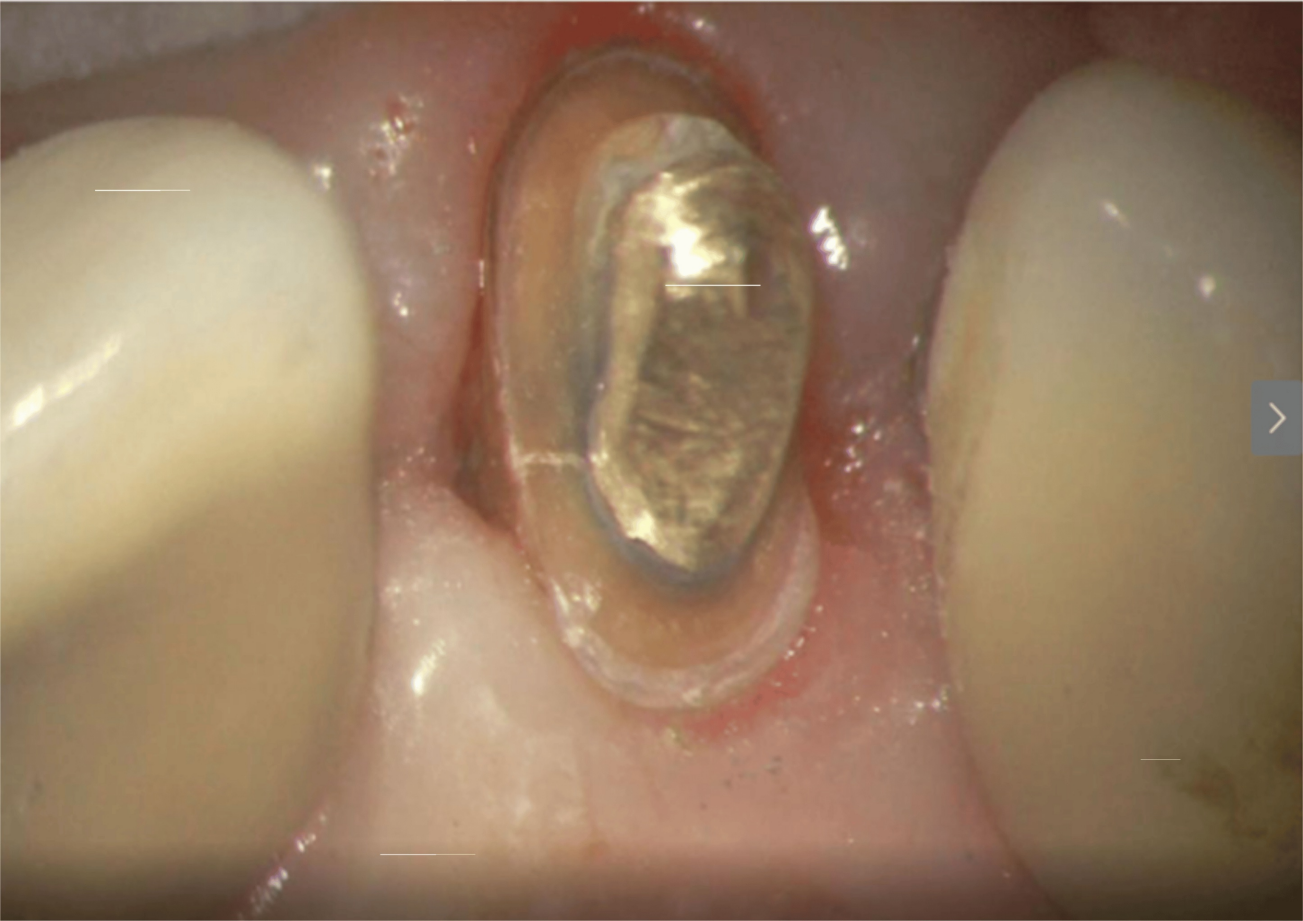
Following removal of the current crown a vertical fracture on the mesial and distal adjacent to the cast post/core

**Figure 23:**
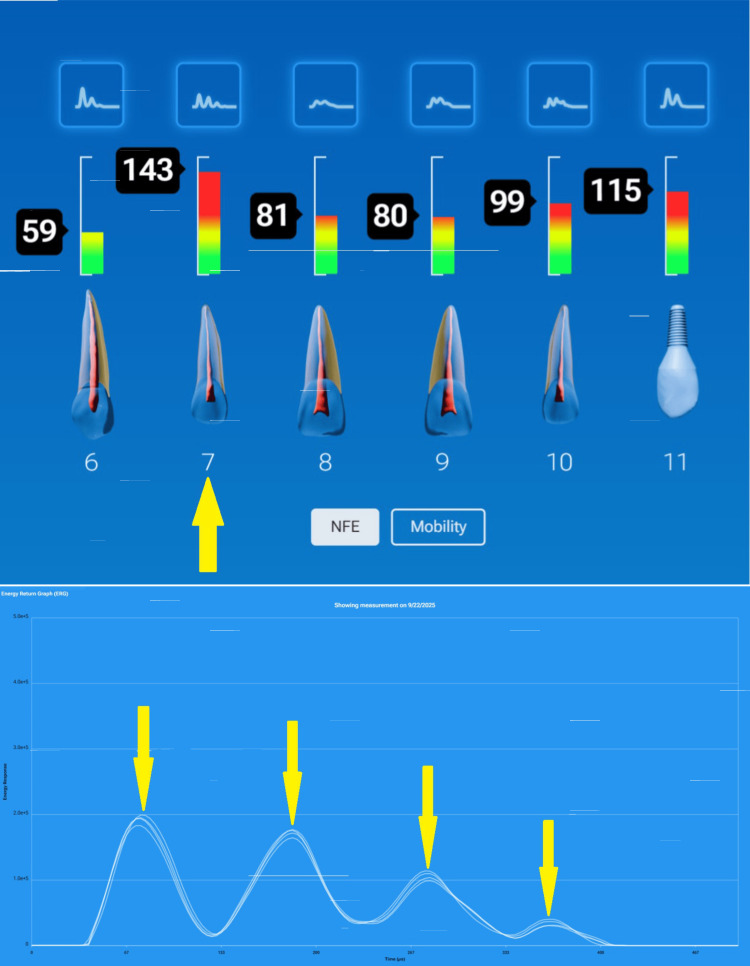
The energy return graph indicated a very structurally unsound tooth with multiple peaks (4) oscillating over a long-time span

However, the pretreatment InnerView test showed normal mobility (Figure 20) but very high NFE (145) on tooth #7 (Figure 21). When the crown was removed, a loose cast gold post/core with a vertical fracture on the mesial and distal proximal surfaces of the tooth was revealed (Figure 22). The ERG demonstrated a very structurally unsound tooth with multiple peaks oscillating over a long-time span (Figure 23). The patient was informed of the vertical root fracture caused by the loose cast post/core, and the recommended treatment was extraction and placement of an implant. Had the periodontist had this QPD information, pocket elimination surgery would not have been performed, and the patient would have been spared the expense and discomfort. 

## Discussion

Objective assessment of structural integrity remains a fundamental unmet need in restorative and implant dentistry. While modern materials and imaging have advanced dramatically, diagnostic methods for detecting early biomechanical failure have lagged, relying largely on indirect, subjective, or late-stage indicators. Fatigue failure of teeth, restorations, and implants is a progressive mechanical process, often occurring well before clinical symptoms or radiographic changes become apparent [11]. By the time failure is visible radiographically or clinically, irreversible structural damage has frequently already occurred, necessitating more invasive intervention [11].

QPD™ addresses this diagnostic gap by applying principles long established in mechanical engineering and structural health monitoring. The ability to measure energy dissipation, oscillatory behavior, and micromobility provides objective insight into how teeth, restorations, and implants respond to functional loading. Those principles parallel nondestructive testing methods used in aerospace and civil engineering to detect microcracks, joint instability, and material fatigue prior to catastrophic failure [12]. This approach in dentistry allows identification of early adhesive degradation, crack initiation, or compromised osseointegration before clinical consequences emerge, allowing early intervention to limit negative outcomes.

Conventional diagnostic tools remain indispensable but are inherently limited. Periapical radiographs and bitewings provide only two-dimensional representations of complex three-dimensional structures and are insensitive to early internal defects or micromovement [3]. Crack visualization is highly dependent on orientation and severity, often rendering early fractures radiographically invisible. Even CBCT imaging, despite its volumetric capability, lacks sufficient spatial resolution to reliably detect fine cracks or early adhesive breakdown and is impractical for routine longitudinal monitoring due to cumulative radiation exposure [13]. Comparative work has shown that CBCT and conventional radiography can miss fine internal defects, reinforcing the need for complementary biomechanical diagnostics such as QPD [14].

Similarly, clinical percussion tests, tactile evaluation, and patient-reported symptoms are subjective and poorly reproducible. RFA provides useful information regarding implant stability but is limited to a single parameter and does not assess the internal structural integrity of restorations or natural teeth [15]. As a result, clinicians are often forced into reactive rather than preventive treatment strategies.

The distinction between overall mobility and internal mobility is critical clinically. Overall mobility reflects bone density, quantity, and osseointegration quality, while internal mobility reflects defects within the tooth-restoration or implant-prosthetic complex. Studies on cracked teeth and failed restorations consistently demonstrate that microcrack propagation and adhesive fatigue precede overt fracture or debonding [16]. The NFE metric captures those subtle internal oscillations, offering a quantifiable indicator of early failure mechanisms.

Early detection of internal instability allows the clinician to intervene conservatively, rather than resorting to crowns, endodontic therapy, or extraction after catastrophic breakdown. This approach aligns with contemporary minimally invasive dentistry principles and preservation of tooth structure.

Implant dentistry similarly benefits from early biomechanical monitoring. Osseointegration is a continuum influenced by bone quality, loading protocols, occlusion, and parafunctional habits [17,18]. While radiographs confirm implant bone contact, they provide no information regarding functional stability under load. Mechanical overload has been strongly associated with crestal bone loss, screw loosening, component fracture, and peri-implantitis progression [19]. This article is a narrative review of QPD and its applications in clinical practice in improving diagnostics that are not evident radiographically or with standard examination methods [20].

Tracking mobility trends over time provides insight into biomechanical stress before inflammatory or radiographic signs develop. Subtle increases in mobility may reflect occlusal overload, bruxism, or prosthetic misfit, allowing early intervention through occlusal adjustment or protective appliances. This proactive approach is consistent with evidence demonstrating that mechanical stability plays a critical role in long-term implant survival and peri-implant health [21].

Quantitative data significantly enhances patient communication and informed consent. Visual representation of ERGs and numerical trendlines transforms abstract concepts of “stability” and “risk” into understandable metrics for the patient. Studies in healthcare decision-making demonstrate that objective visual data improve patient understanding, trust, and treatment acceptance [22]. This is particularly relevant when recommending preventive or proactive care for asymptomatic conditions. From a workflow perspective, the ability to perform full-mouth testing in under two minutes allows seamless integration into new-patient examinations and hygiene recall visits. Longitudinal monitoring establishes a structural health baseline, analogous to periodontal charting, enabling clinicians to track biomechanical changes over time rather than relying on isolated snapshots.

As with any diagnostic modality, QPD™ is operator-dependent, and consistency in tip placement and technique is essential for reproducibility. Built-in deviation alerts mitigate variability, but appropriate training remains necessary. Importantly, QPD™ is not intended to replace radiographs or clinical examination but to complement them by providing biomechanical data that other modalities cannot.

## Conclusions

InnerView® and QPD™ represent a significant advance in diagnostic capabilities for both teeth, implants, and their restorations. This is based on decades of research from broad interdisciplinary teams of dentists, engineers, data scientists, and statisticians. By quantifying micromobility, internal damping, and subtle structural variations, the technology can identify early biomechanical and adhesive failures that traditional methods cannot detect. Compared with standard diagnostics, which often confirm disease only after it has occurred, QPD™ enables true prevention with early detection and minimally invasive care. Incorporating this objective data into clinical workflows enhances treatment planning, which improves long-term outcomes, ultimately elevating the standard of care in restorative and implant dentistry.
